# The impact of COVID-19 stressors on psychological distress and suicidality in a nationwide community survey in Taiwan

**DOI:** 10.1038/s41598-022-06511-1

**Published:** 2022-02-17

**Authors:** Chia-Yi Wu, Ming-Been Lee, Pham Thi Thu Huong, Chia-Ta Chan, Chun-Yin Chen, Shih-Cheng Liao

**Affiliations:** 1 School of Nursing, National Taiwan University College of Medicine, Taipei, Taiwan; 2 Department of Nursing, National Taiwan University Hospital, Taipei, Taiwan; 3 Taiwanese Society of Suicidology & Taiwan Suicide Prevention Center, Taipei, Taiwan; 4 Department of Psychiatry, Shin Kong Wu Ho-Su Memorial Hospital, Taipei, Taiwan; 5 Department of Psychiatry, National Taiwan University College of Medicine, Taipei, Taiwan; 6 Faculty of Nursing and Midwifery, Hanoi Medical University, Hanoi, Vietnam; 7 National Institute of Mental Health, Bach Mai Hospital, Hanoi, Vietnam; 8 Department of Psychiatry, National Taiwan University Hospital, Taipei, Taiwan; 9 School of Nursing, National Taiwan University College of Medicine, 1, Jen-Ai Road Section 1, Taipei, 10051 Taiwan

**Keywords:** Health care, Risk factors

## Abstract

COVID-19 stressors and psychological stress response are important correlates of suicide risks under the COVID-19 pandemic. This study aimed to investigate the prevalence of COVID-19 stress, its impact on mental health and associated risk factors among the general population during the outbreak of COVID-19 in July 2020 throughout Taiwan. A nationwide population-based survey was conducted using a computer-assisted telephone interview system with a stratified, proportional randomization method for the survey. The questionnaire comprised demographic variables, psychological distress assessed by the five-item Brief Symptom Rating Scale and independent psychosocial variables including COVID-19 stressors, loneliness, suicidality, and health-related self-efficacy. In total, 2094 respondents completed the survey (female 51%). The COVID-19 stress was experienced among 45.4% of the participants, with the most prevalent stressors related to daily life and job/financial concerns. Higher levels of suicidality, loneliness, and a lower level of self-efficacy had significantly higher odds of having COVID-19 stress. The structural equation model revealed that COVID-19 stress was moderately associated with psychological distress and mediated by other psychosocial risk factors. The findings call for more attention on strategies of stress management and mental health promotion for the public to prevent larger scales of psychological consequences in future waves of the COVID-19 pandemic.

## Introduction

Since the outbreak of the novel coronavirus (COVID-19) in March 2020^1^, Taiwan was assumed to be the second-highest country of confirmed COVID-19 cases due to the close distance to mainland China^2^^.^ However, Taiwan has survived the global COVID-19 impacts in 2020 without lockdown as in many other countries^3^; instead, most people in Taiwan followed government precautions with care under quick responses of health services and transparency of information^4^. The spread of COVID-19 and the resulting economic recession has increased major psychological health problems worldwide, which resulted in 25% to 50% of the population experiencing mental health impacts during the pandemic^5,6^. A high level of loneliness, worries about the pandemic, and low distress tolerance were reported and significantly associated with clinical levels of depression, anxiety, and post-traumatic stress disorder symptoms^7–9^. Further, the overall pooled prevalence of abovementioned psychological responses ranged between 30 and 40% across countries in a systematic review^5^. In Taiwan, the “Mood Thermometer” application (also called the five-item Brief Symptom Rating Scale, BSRS-5) was promoted during COVID-19 as a self-care tool with high-tech assistance linking psychological distress assessment and resources referrals^10^. The BSRS-5 self-rating scale represents the assessment of psychopathology including insomnia, anxiety, hostility, depression, and inferiority respectively. However, the evidence of the population effect is limited regarding the public psychological responses toward perceived stress of the general public under COVID-19. Further, it is unknown whether the most severe form of psychological health problem- suicidality would be related to COVID-19-related stress and mental distress. Given the cutting-edge role of pharmacological approach in suicide prevention such as ketamine treatment^11^, there is also an urgent need to examine the global environmental factors of stressors and their associations with suicide during the pandemic. Suicidality can be conceptualized as suicidal behaviors including suicide ideation and attempts at different time^12^.

Recent studies reported a significant association between self-efficacy and psychological factors during the COVID-19 outbreak^13,14^. The lower level of self-efficacy indicates being unable to manage the situation effectively, even though a person knows what to do or having the requisite skills^13^. One study about severe acute respiratory syndrome (SARS) revealed that direct experience of SARS led to higher levels of self-efficacy, while the lower level of self-efficacy may lead to a lack of protective motivation^15^. In addition, self-efficacy was recognized as an important component in promoting health-related intention and behavior change^16,17^, and it was also related to an inappropriate belief of personal ability and capacity to cope with COVID-19^12^. Further, health-related self-efficacy was conceptualized as a binary measure associating with lifetime suicidal ideation, prior suicidal attempts, and future suicide intent^18^. Only a few studies have focused on exploring the association between self-efficacy and suicidal behavior. Hence, the need for health-related self-efficacy assessment would be an important target for studies of psychological health including those with suicide risk assessment.

The COVID-19 outbreak has led to various restriction policies that interfere economic growth across all sectors with increasing rates of unemployment, financial insecurity, as well as poor mental health or suicide risks^19,20^. The negative outcomes of COVID-19 indicated that the pandemic has led to unprecedented hazards and impacts that are more than health or mental health crises alone^21^. Specifically, the economic recession was positively associated with a higher suicide rate compared with the period of prosperity^22,23^. Recent surveys showed that the COVID-19 related mental health impact might not be prominent during the outbreak; however, those who were jobless or under financial issues might be the target for follow-ups in long-term suicide prevention strategies^20,21^. In this study, the concept of COVID-19 stressors refers to six domains of stressors over the past month during the COVID-19 pandemic, including physical health, mental health, family/interpersonal relationships, work/financial, schooling, and daily life.

This nationwide population-based survey aimed to investigate the prevalence of psychiatric morbidity and the associations between COVID-19 stressors, psychological distress and suicidality in Taiwan. The psychological distress and personal experience with COVID-19 stressors, suicidality, loneliness, and health related self-efficacy were examined in a representative sample of Taiwan. In this study the measurement of psychological distress was conceptually termed as psychiatric morbidity and measured by the same scale, so the two terms were used interchangeably.

## Methods

### Study setting and data collection

The study used a computer-aided telephone interview method to recruit a representative sample in Taiwan during July 2020, which was 4 months after the WHO declared COVID-19 outbreak a global pandemic. In this survey, the landline telephone numbers were randomly selected via stratified proportional sampling based on the distribution of population size, gender, and age in different geographic areas of Taiwan. The study was approved by the Institutional Review Board (IRB) in National Taiwan University Hospital (reference number: 202103109W). All respondents and/or their legal guardians provided informed consent and were being assured of anonymity and confidentiality. The study was performed in accordance with relevant guidelines regulated in the above-mentioned IRB. All the data were collected by well-trained interviewers over the telephone.

### Participants

All participants were aged ≥ 15 years old and agreed to participate in the survey anonymously over the phone and accomplished the interview (with a sampling error of + 2.10% in 95% confidence interval). The method and procedure of participant recruitment was also described elsewhere^24^. The population-based study sampling was performed via the project administrator of the Taiwan Suicide Prevention Centre (TSPC). Specifically, participants were contacted upon sampling via a telephone survey on population mental health, knowledge and behavior related to suicide prevention.

### Measurements

#### Psychological distress

The BSRS-5 was used to measure the level of psychological distress in the past week of the respondents^25,26^. It is a 5-point Likert scale (0–4) that contains the following questions: (1) having trouble falling asleep (insomnia); (2) feeling tense or keyed up (anxiety); (3) feeling easily annoyed or irritated (hostility); (4) feeling low in mood (depression), and (5) feeling inferior to others (inferiority). An additional question for assessment of recent suicide ideation “Do you have any suicide ideation in the past week?” was added at the end of the scale. The BSRS-5 has satisfactory psychometric properties to detect psychiatric morbidity and recent suicide ideation in medical settings or the community^27,28^. In this study, the presence of psychiatric morbidity was defined by BSRS-5 with a score of $\ge$ 6 or greater. The internal consistency of the BSRS-5 in this study was satisfactory (Cronbach’s alpha: 0.80) and comparable with the previous study (0.89)^24^.

#### COVID-19 stressors

All the participants were asked whether they experienced any of the following six domains of stressors over the past month during the COVID-19 pandemic, including physical health, mental health, family/interpersonal relationships, work/financial, schooling, and daily life. All the domains of questions reflected self-report stress perceptions of life or personal health conditions, e.g., physical health refers to general well-being judged out of a person’s own perception. These domains were designed to reflect typical stress sources under the COVID-19 crisis. Response options were “Yes” (1 point) or “No” (0 point). Participants who reported “Yes” in each domain of COVID-19 stressors were classified as the presence of COVID-19 stress. Due to the very low response in the variable of school-related stress, it was excluded in most tables.

#### Suicidality

We evaluated whether the respondents had previous history of suicide attempt/ ideation across different time points, including past 1-week, past 1-month, past 1-year, and in lifetime. Moreover, the item of future suicide intent was also assessed with the question, “Do you intent to harm yourself or attempt suicide in the future?” The above items were screening questions drawing responses of “Yes” or “No”.

#### Health-related self-efficacy

A single-item question was used to assess health-related self-efficacy^29^. The participants were asked, “How much confidence, from a scale of 0 to 100, do you think you have control over your own health conditions?”^30^. The higher the score, the better the confidence of their health control. It was divided into three categories by tertile (i.e., low = 0–79, moderate = 80–85, and high = 86–100).

#### Feelings of loneliness

Loneliness was assessed using a single general question for screening, "Do you often feel lonely?" (1 = yes, 0 = no). The single-item format of measurement was used in large-scale national surveys previously^31,32^. The variable was shown to be a valid predictor for both physical and mental distress including depression, anxiety, and suicide risks^32^.

### Statistical analysis

Data were analyzed after weighting for age and gender by the raking weighting method to make the sample best represent the entire general population. In addition to descriptive statistics of demographic variables, the chi-square test was applied to examine the associations between COVID-19 stress and suicidality, feelings of loneliness, psychiatric morbidity, and self-efficacy. Moreover, the Pearson’s correlation was conducted to test the associations between the above-mentioned variables. The graphical relationship was presented between variables of psychological distress and the amount of reported COVID-19 stressors. Finally, the structural equation model was performed to examine the associating factors depicting in this study that predicted COVID-19 stress. Statistical significance was set at a level of p < 0.05.

## Results

### Participant characteristics

In total, 2094 participants were recruited nationwide from the Northern (46%), the Central (27%), Southern (25%), and Eastern (2%) regions of Taiwan, which distribution is reflective of the population ratio. As can be seen in the Table 1, there were 1031 males (49.2%) and 1063 females (50.8%) in this study. The distributions for different age groups as 15–24 (13.3%), 25–44 (34.6%), 45–64 (34.3%) and above 65 (17.8%). The most common COVID-19 stress was related to daily life (24.8%, n = 519) and job/finance (23.4%, n = 490), followed by mental health conditions (18.7%, n = 392), physical health (17.0%, n = 355), family/interpersonal relations (10.9%, n = 227), and schooling (10.2%, n = 23). Overall, nearly half (45.4%) of the participants reported having at least one significant COVID-19 stressor over the preceding month of the survey (Supplementary Table S1).

**Table 1:** Sociodemographic characteristics of the participants (N = 2094).

	n	%
**Gender**		
Male	1031	49.2
Female	1063	50.8
**Age**		
15–24	277	13.3
25–34	324	15.5
35–44	400	19.1
45–54	363	17.3
55–64	355	17.0
65 and above	375	17.8
**Marital status**		
Unmarried	684	32.7
Married	1336	63.8
Divorce	41	2.0
Widowed	30	1.4
Separated	2	0.1
**Education level**		
Elementary	160	7.6
Secondary	194	9.3
High school	599	28.6
Vocational degree	257	12.3
University and above	882	42.1
**Regions in Taiwan**		
Northern	964	46.0
Central	566	27.0
Southern	524	25.0
Eastern	40	2.0

All the above-mentioned data were weighted. The data of no response were excluded for analysis.

### Demographics and COVID-19 stress

No significant gender difference was found for any domain of COVID-19 stressors except for the higher prevalence of physical health stress (19.5% females vs 14.4% males) (p = 0.002). The age-subgroup analysis showed a significant difference in 4/5 domain of COVID-19 stressors, apart from daily life stress. While those from 25 to 64 years old had the highest prevalence in job/financial stress ranging from 26.4 to 29.2%, respondents over 65 years old reported the most stressors in mental health and family/interpersonal aspects (24% and 14.7%) (p < 0.001). Physical health stressor was experienced two times higher among those from 25 to 39 years old and over 65 years old (21.9% and 20.3% respectively) than those from 15 to 24 years old (11.6%). Among the young respondents under schooling age, only 23 students reported stress related to schooling, and the majority was from the 15–24 age groups (82.6%). In terms of marital status and COVID-19 stress, the widowed group appeared to be the most vulnerable by showing statistically significant difference in daily life stressors (46.7%), job/financial issues (30.0%), and family/interpersonal relationship (20.0%). Especially, job/financial stress was the most prevalent among those in the divorce, widowed, and separated groups (p < 0.05). Concerning the association between COVID-19 stressors and occupation, all the differences appeared statistically significant except the schooling variable. While shop owners/business investors reported significantly perceived stress in job/finance (49.5%) and mental health (27.8%) areas, the occupation of professionals was the majority in experiencing stress related to daily life (38.6%) and physical health (31.8%). Housewives experienced relatively higher family/interpersonal stress (15%) than their counterparts. In addition, compared to a government employee, shop owners or business investors had an 8.5-time higher risk of experiencing job/financial stress under COVID-19 outbreak (Supplementary Tables S2–4).

### Psychological distress, suicidality, and COVID-19 stress

Table 2 illustrates the independent association between suicidality, psychiatric morbidity and other psychosocial variables by the presence of COVID-19 stress. Almost all variables appeared significantly correlated with the presence of COVID-19 stressors except the suicide attempt in 1 year and 1 month. In total, 253 (12.1%) reported having lifetime suicide ideation and 45 (2.2%) reported having suicidal ideation in the past year; 40 (1.9%) reported having lifetime suicide attempt and 3 (0.1%) attempted suicide in the past year, with future suicide intent reported by 29 (1.4%) participants. Those who reported suicidal ideation in the recent 1 month had an association with 10 times higher likelihood of suffering from any COVID-19 stress compared to their counterparts (OR = 10.310, 95% CI 2.325–3.889). Moreover, the risk of suicide ideation in the past 1 year (OR = 3.716, 95% CI 1.886–7.320), suicide ideation in the past week (OR = 2.685, 95% CI 1.137–6.341), future intent to suicide (OR = 2.348, 95% CI 1.093–5.044), lifetime suicide ideation (OR = 2.324, 95% CI 1.770–3.050), and lifetime suicide attempt (OR = 2.044, 95% CI 1.074–3.889) were correlated with the presence of COVID-19 stressors. People with feelings of loneliness were associated with higher odds of perceiving COVID-19 stress (OR = 3.248, 95% CI 2.421–4.357). Specifically, the risk of the presence of COVID-19 stress was the highest (OR = 4.696, 95% CI 3.041–7.252) for those with psychiatric morbidity. Low and moderate health-related self-efficacy also showed an association with COVID-19 stress. The odds for experiencing COVID-19 stress among those with low and moderate health-related self-efficacy were 0.371 (95% CI 0.295–0.465) and 0.656 (95% CI 0.529–0.814) respectively.

**Table 2:** The odds of COVID-19 stress by suicidality, psychiatric morbidity and psychosocial variables.

n (%)	COVID-19 stressors		Total	p-value	OR(95%CI)
	Presence	Absence			
**Lifetime suicide ideation**				< 0.001	
Yes	161 (16.9)	92 (8)	253 (12.1)		2.324 (1.770–3.050)
No	789 (83.1)	1051 (92)	1840 (87.9)		
**Suicide ideation in 1 year**				< 0.001	
Yes	34 (3.6)	11 (1)	45 (2.2)		3.716 (1.886–7.320)
No	916 (96.4)	1132 (99)	2048 (97.8)		
**Suicide ideation in 1 month**				< 0.001	
Yes	16 (1.7)	2 (0.2)	18 (0.9)		10.310 (2.325–45.716)
No	933 (98.3)	1141 (99.8)	2074 (99.1)		
**Lifetime suicide attempt**				0.028	
Yes	25 (2.6)	15 (1.3)	40 (1.9)		2.044 (1.074–3.889)
No	925 (97.4)	1129 (98.7)	2054 (98.1)		
**Suicide attempt in 1 year**				0.676	
Yes	1 (0.1)	2 (0.2)	3 (0.1)		0.554 (0.033–9.210)
No	949 (99.9)	1143 (99.8)	2092 (99.9)		
**Feelings of loneliness**				< 0.001	
Yes	167 (17.6)	70 (6.1)	237 (11.3)		3.248 (2.421–4.357)
No	783 (82.4)	1072 (93.9)	1855 (88.7)		
**Future intent to suicide**				0.026	
Yes	19 (2)	10 (0.9)	29 (1.4)		2.348 (1.093–5.044)
No	912 (98)	1122 (99.1)	2034 (98.6)		
**BSRS-5 scores**				< 0.001	
< 6 (mental well-being)	853 (89.8)	1117 (97.6)	1970 (94.1)		4.696 (3.041–7.252)
$\ge$ 6 (mental distress)	97 (10.2)	27 (2.4)	124 (5.9)		
**Health self-efficacy**				< 0.001	
0–79	367 (39.5)	266 (23.5)	633 (30.7)		0.37 (0.295–0.465)
80–85	345 (37.1)	443 (39.1)	788 (38.2)		0.656 (0.529–0.814)
86–100	217 (23.4)	424 (37.4)	641 (31.1)		

All the above-mentioned data were weighted; missing data were excluded for analysis.

BSRS-5: The Five-item Brief Symptom Rating Scale; OR: odds ratio; CI: confidence interval.

**Figure 1:**
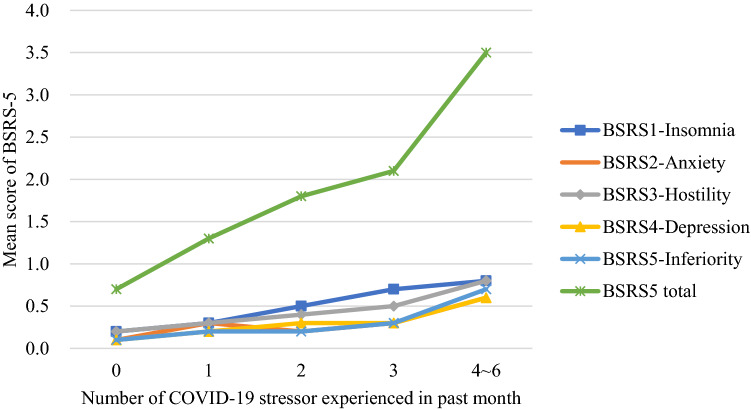
The association between psychological distress and the amount of reported COVID-19 stressors.

The pattern for the association between psychological distress and COVID-19 stressors was revealed in Fig. 1. The result showed that the more COVID-19 stressors the participants experienced, the higher score of psychological distress they suffered in the past month. An overall increase in all five psychopathology symptoms (e.g., insomnia, anxiety, hostility, depression, and inferiority) is inevitable since the number of COVID-19 stressors is rising, leading to a negative effect on performance in the total score of BSRS-5 (p < 0.001).

### Self-efficacy, psychological distress, and COVID-19 stress

As reported in Table 3, five domains of COVID-19 stressors and health-related self-efficacy were significant risk factors linked to psychiatric morbidity. Among COVID-19 stressors, family/interpersonal stressor was linked to psychiatric morbidity in aggravating a respondent (OR = 5.821, 95% CI 3.923–8.637), followed by mental health (OR = 3.793, 95% CI 2.612–5.507), job/financial hardship (OR = 3.332, 95% CI 2.307–4.811), and physical health (OR = 2.849, 95% CI 1.933–4.198). Moreover, we found that the participants in the low and moderate health-related self-efficacy category had about 7.5 times (OR = 7.419, 95% CI 3.895–14.132) and 2.8 times (OR = 2.834, 95% CI 1.434–5.599) respectively higher likelihood of presenting psychiatric morbidity than those in the high health-related self-efficacy category.

**Table 3:** The odds of  psychiatric morbidity by COVID-19 stressors and health or psychosocial variables.

n (%)	Psychiatric morbidity		Total	p-value	OR(95% CI)
	Absence (BSRS-5 < 6)	Presence (BSRS-5 $\ge$ 6)			
**Physical health**					
Yes	312 (15.8)	44 (35.2)	356 (17)	< 0.001	2.849 (1.933–4.198)
No	1657 (84.2)	81 (64.8)	1738 (83)		
**Mental health**					
Yes	337 (17.1)	55 (44)	392 (18.7)	< 0.001	3.793 (2.612–5.507)
No	1633 (82.9)	70 (56)	1703 (81.3)		
**Stress of family/interpersonal relations**					
Yes	181 (9.2)	46 (37.1)	227 (10.8)	< 0.001	5.821 (3.923–8.637)
No	1789 (90.8)	78 (62.9)	1867 (89.2)		
**Stress of job/financial trouble**					
Yes	430 (21.8)	60 (48.4)	490 (23.4)	< 0.001	3.332 (2.307–4.811)
No	1539 (78.2)	64 (51.6)	1603 (76.6)		
**Stress related to daily life**					
Yes	457 (23.2)	61 (49.2)	518 (24.7)	< 0.001	3.204 (2.221–4.623)
No	1512 (76.8)	63 (50.8)	1575 (75.3)		
**Self-efficacy**					
Low (0–79)	560 (28.9)	73 (60.3)	633 (30.7)	< 0.001	7.419 (3.895–14.132)
Moderate (80–85)	751 (38.7)	37 (30.6)	788 (38.2)	0.0027	2.834 (1.434–5.599)
High (86–100)	630 (32.5)	11 (9.1)	641 (31.1)		

All the above-mentioned data were weighted; missing data were excluded for analysis.

BSRS-5: The Five-item Brief Symptom Rating Scale; OR: odds ratio; CI: confidence interval.

Supplementary Table S5 illustrates the inter-item associations of COVID-19 stressors, (psychological distress, loneliness, and health-related self-efficacy). All the associations were statistically significant, with Pearson’s rank correlation coefficient ranging from − 0.253 to 0.493. Especially, all five COVID-19 stressors had the strongest correlation with psychological distress, with the correlation coefficient ranged from 0.205 to 0.275, followed by loneliness (from 0.132 to 0.198), and health-related self-efficacy (from − 0.156 to − 0.114).

### Modeling factors predicting COVID-19 stress

**Figure 2:**
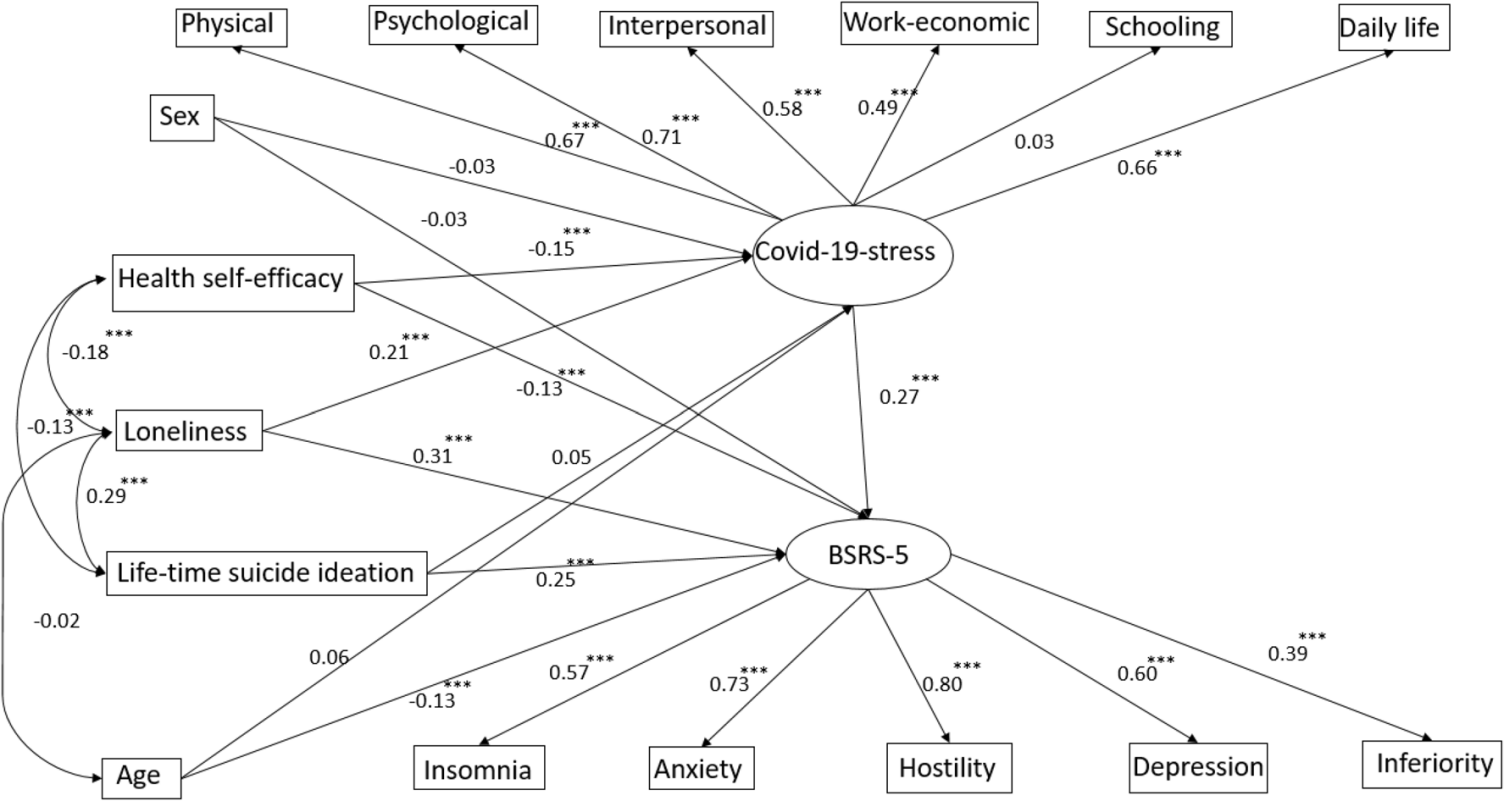
The structural equation model for psychological distress by COVID-19 stressors with control for related psychosocial factors. Root mean square error of approximation (RMSEA) = 0.047 < 0.05, goodness of fit index (GFI) = 0.969 > 0.9, adjusted goodness of fit index (AGFI) = 0.954, Cmin/DF = 5.607, p < 0.0001.

Moreover, the structural equation model on the predictive validity of the above significant psychosocial correlates of COVID-19 stressors as intermediate independent variables for psychological distress revealed a satisfactory adjusted goodness-of-fit value of 0.954 (p < 0.001), which indicated that the model approximated the real structure as shown in Fig. 2. As predicted by the model, the regression correlations between each variable were demonstrated. Among all the variables, loneliness had a more direct effect on COVID-19 stress ($\beta$ = 0.21), psychological distress ($\beta$ = 0.31) and lifetime suicide ideation ($\beta$ = 0.29). The COVID-19 stressors had a relatively higher impact on psychological distress with the direct association, with the coefficient 0.27 in the model.

## Discussion

This population-based survey investigated COVID-19 pandemic stressors with the correlates of psychological distress and suicidality under the outbreak in July 2020. About half of the representative sample suffered at least one significant COVID-19 stressor, mainly on two common sources of daily life and job/financial problems. Characteristics of lifetime suicide ideation/attempt, suicide ideation during the last year/month/week, future suicide intent, lower self-efficacy and loneliness were significantly attributable to COVID-19 stress. Specificity, loneliness was directly associated with various mental health related variables including psychological distress, COVID-19 stress, and lifetime suicide ideation. These findings add to the literature that supports a relationship between the impact of COVID-19 stressors on psychological distress and suicide risk in the Taiwanese general population.

Overall, daily life stress and job/financial issue were found to be the most common stressors among the Taiwanese general population toward the impact of COVID-19. When COVID-19 has posed major challenges to the public and government, an individual could be affected in many aspects of life, such as socializing, working, studying, living, and lifestyle^8^. This disruption of daily routines can greatly impact mental health during crises^8,33^. The COVID-19 outbreak was reported as a massive reduction to economic activities globally, many industries especially tourism and food sectors were among the worst-hit service in Taiwan, which negatively impact the whole society^34^. As a result, more people experienced reducing salaries and unemployment throughout the pandemic. According to the Taiwanese Labor Insurance Bureau, the number of people with unemployment increased sharply to 23.75% compared to the number of 2019^34^. Similarly, a survey conducted during June 2020 in Taiwan showed respondents worried about losing jobs and financial hardships than their mental health issues^21^. This is consistent with global literature suggesting that the uncertainty of financial impact and the unemployment rate can put individuals at greater risks for developing psychological distress and adverse mental disorders during the pandemic^12^.

In our sample, the lifetime, past-year, past-month, and past-week suicide ideation were prevalent at 12.1%, 2.2%, 0.9%, and 1.2% of the respondents, whereas 1.9%, 0.1%, and 0 (n = 1) of the sample attempted suicide in the lifetime, past year, and past month, respectively. These results indicated a generally low suicide risks and were consistent with our previous annual survey results in Taiwan, which showed the prevalence of lifetime suicide ideation and lifetime suicide attempt as of 12.6% and 2.7%^18^. Moreover, the rates of recent suicide ideation (1.2%) were much lower compared to the studies in Norwegian (3.6%)^35^, the UK (8.2–9.8%)^36^, and the US (10.7%)^37^. Our result is consistent with the results from a meta-analysis of 21 high- and upper-middle-income countries that reported no change or decline in suicide rates in the early month of the pandemic compared to the expected levels^38^. It is probable that the lower prevalence of suicide in Taiwan after the first wave of COVID-19 was related to the lower number of confirmed cases and deaths than the abovementioned countries. In addition, complications of virus infection and extensive health and economic burden caused by quarantine or other preventive strategies (i.e., isolation, worry about family members and friends, economic concerns) may increase the risk of suicidal thoughts in those countries^38^. A recent analysis of the influenza pandemic during 1918–1920 in Taiwan showed that suicide rates were no higher than expected during the first wave of the outbreak. However, an increase in delayed suicide rate was revealed during the second wave of infection (33–35%)^39^. Similarly, the prevalence of suicide ideation in the UK increased from 8.2% (wave 1: 31 March to 9 April 2020), 9.2% (wave 2: 10 April to 27 April 2020) to 9.8% (wave 3: 28 April to 11 May 2020)^36^ as the pandemic progressed. These findings informed the importance to keep watching the fluctuations of suicide risks in future waves of pandemic for early interventions.

Moreover, our findings showed that COVID-19 stressors directly predicted psychological distress in the structural equation model, and loneliness was positively associated with lifetime suicide ideation, COVID-19 stress, and psychological distress. Our result is in line with recent findings from Brazil, in which a significant association between self-report loneliness and suicidal ideation was salient during the pandemic^40^. This result suggests the need of long-term care for those living with suicide ideation under COVID-19, especially those with history of suicide ideation, suicide attempts or completed suicide^41,42^. Notably, there is robust evidence in the literature regarding the increasing vulnerability to mood disorders and suicide due to influenza infection^43^. Therefore, it is logical to assume that an increasing trend of suicide may happen due to COVID-19 stressors at later stages of the pandemic, calling for more preventive strategies and lasting solutions prioritized by state policymakers, government agencies, non-profit organizations, and health care professionals^44^. To continue and strengthen the implementation of suicide prevention strategies during and after the pandemic, early detection, proactive prevention, and longer-term control measurements of the risk factors of suicide are highly suggested.

The study demonstrated an increased risk of psychological distress and COVID-19 stress among people with lower health-related self-efficacy levels. Self-efficacy was proposed as a protective factor against psychological distress under COVID-19 pandemic and a sense of personal control over behavioral changes^45,46^. The underlying study supported the role of self-efficacy in the association between stress and mental distress. Our previous study further identified the association between self-efficacy and suicide risks across different timeframe^47^. Future investigations of self-efficacy facilitation and its longitudinal observations with suicide risks under stress will be needed to develop proper management for suicide high-risks during the pandemic.

Interpretation of the study should be cautious due to several limitations. First, the cross-sectional design may limit the causal inference of the study. Second, the telephone interview method might restrict to the people who did not use the landlines frequently, and those who refused to respond were not possibly recorded by age and gender, thus limited our comparison of non-responders and responders. However, the landline telephone is considered one of the best ways to approach the participants staying at home during COVID-19, so we have used a relatively feasible way of recruitment. Third, the interview may be affected by the respondent’s surroundings which the researcher has limited control. However, experienced interviewers with guideline questionnaires and standard operating procedures developed by the researcher team and TSPC could ensure high quality and reliable data when approaching the respondents.

Despite the limitations, several key strengths should be noted in our study. First, the underlying surveys have been conducted by TSPC annually since 2006 with a large sample size randomly selected, ensuring the representativeness of the whole country. Second, the well-trained telephone interviewer could reduce the complexity and sensitivity of the suicidality topic. The respondents could answer comfortably and feel relaxed and able to disclose sensitive information via telephone due to anonymity. Finally, evidence-based findings based on previous publications of this nationwide annual surveys have provided robust evidence in the methodology^18,24^. The finding from this study might help relevant stakeholders in designing and implementing multisectoral approaches to provide adequate interventions for individuals at risk of psychological distress and suicide.

## Conclusions

In this community-based survey, half of the respondents reported experiencing at least one recent stressor during the COVID-19 pandemic, in which daily life, job/financial, and family/interpersonal related stress were the commonest concerns. These stress perceptions were significantly correlated with suicidality, loneliness, and self-efficacy with notable odds ratios. Multiple psychosocial risk factors were attributable to COVID-19 stress, including loneliness, psychological distress, lifetime suicide ideation, and self-efficacy. The present study provides initial evidence that informs future research and policy development about mental health promotion strategies during COVID-19 among the general public. In addition, COVID-19 stress-related risk factors should receive timely attention, particularly on stress management strategies in community mental health services given potential outbreaks of COVID-19 ahead globally.

## Supplementary Information

Supplementary Tables.
