# Supplementary material for: The impact of COVID-19 stressors on psychological distress and suicidality in a nationwide community survey in Taiwan
Source: Sci Rep. 2022 Feb 17;12:2696. doi: 10.1038/s41598-022-06511-1 (PMC8854558; doi:10.1038/s41598-022-06511-1)
Supplement: Supplementary file 1 — Supplementary Tables. [file 41598_2022_6511_MOESM1_ESM.docx]

**Table S1**. Reported COVID-19 related stressors by gender (N=2094)

| Stressors | Male | Female | Total | p-value |
| --- | --- | --- | --- | --- |
|  | n (%) | n (%) | N (%) |  |
| Physical health |  |  |  | 0.002 |
| Yes | 148 (14.4) | 207 (19.5) | 355 (17) |  |
| No | 880 (85.6) | 856 (80.5) | 1736 (83) |  |
| Mental health |  |  |  | 0.066 |
| Yes | 176 (17.1) | 215 (20.2) | 391 (18.7) |  |
| No | 854 (82.9) | 848 (79.8) | 1702 (81.3) |  |
| Family/interpersonal relations |  |  |  | 0.804 |
| Yes | 110 (10.7) | 117 (11) | 227 (10.9) |  |
| No | 919 (89.3) | 944 (89) | 1863 (89.1) |  |
| Job/financial trouble |  |  |  | 0.125 |
| Yes | 256 (24.9) | 234 (22) | 490 (23.4) |  |
| No | 774 (75.1) | 829 (78) | 1603 (76.6) |  |
| Schooling-related |  |  |  |  |
| Yes | 12 (9.9) | 11 (10.6) | 23 (10.2) | 0.871 |
| No | 109 (90.1) | 93 (89.4) | 202 (89.8) |  |
| Daily life |  |  |  | 0.423 |
| Yes | 247 (24) | 271 (25.5) | 518 (24.8) |  |
| No | 782 (76) | 791 (74.5) | 1573 (75.2) |  |
| BSRS-5 levels |  |  |  | 0.045 |
| 0-5 | 983 (95.4) | 986 (92.8) | 1969 (94.1) |  |
| 6-9 | 33 (3.2) | 46 (4.3) | 79 (3.8) |  |
| 10-14 | 13 (1.3) | 29 (2.7) | 42 (2) |  |
| 15 -20 | 1 (0.1) | 2 (0.2) | 3 (0.1) |  |
| Note: All the above-mentioned data were weighted; The data of no response were excluded for analysis. | | | | |

**Table S2.** Reported COVID-19 stressors by age (N = 2094)

| Stressors |  | | | | |  |
| --- | --- | --- | --- | --- | --- | --- |
|  | 15-24 | 25-39 | 40-64 | 65 | Total | p-value |
|  | n (%) | n (%) | n (%) | n (%) | N (%) |  |
| Physical health |  |  |  |  |  | <0.001 |
| Yes | 32 (11.6) | 106 (21.9) | 142 (14.9) | 76 (20.3) | 356 (17) |  |
| No | 245 (88.4) | 378 (78.1) | 813 (85.1) | 299 (79.7) | 1735 (83) |  |
| Mental health |  |  |  |  |  | <0.001 |
| Yes | 26 (9.4) | 90 (18.5) | 186 (19.5) | 90 (24) | 392 (18.7) |  |
| No | 251 (90.6) | 397 (81.5) | 769 (80.5) | 285 (76) | 1702 (81.3) |  |
| Family/interpersonal relations |  |  |  |  |  | <0.001 |
| Yes | 10 (3.6) | 56 (11.5) | 106 (11.1) | 55 (14.7) | 227 (10.9) |  |
| No | 265 (96.4) | 429 (88.5) | 850 (88.9) | 319 (85.3) | 1863 (89.1) |  |
| Job/financial trouble |  |  |  |  |  | <0.001 |
| Yes | 29 (10.5) | 142 (29.2) | 252 (26.4) | 67 (17.9) | 490 (23.4) |  |
| No | 248 (89.5) | 345 (70.8) | 703 (73.6) | 307 (82.1) | 1603 (76.6) |  |
| Schooling |  |  |  |  |  | 0.012 |
| Yes | 19 (9) | 4 (30.8) | - | - | 23 (10.2) |  |
| No | 193 (91) | 9 (69.2) | - | - | 202 (89.8) |  |
| Daily life |  |  |  |  |  | 0.089 |
| Yes | 53 (19.1) | 133 (27.3) | 238 (24.9) | 95 (25.5) | 519 (24.8) |  |
| No | 224 (80.9) | 354 (72.7) | 717 (75.1) | 278 (74.5) | 1573 (75.2) |  |

Note: All the above-mentioned data were weighted; The data of no response were excluded for analysis.

**Table S3.** Reported stressors by marital status (N = 2094)

|  | Unmarried | Married | Divorce | Widowed | Separated | Total | p-value |
| --- | --- | --- | --- | --- | --- | --- | --- |
|  | n (%) | n (%) | n (%) | n (%) | n (%) | N (%) |  |
|  |  |  |  |  |  |  |  |
| Physical health |  |  |  |  |  |  | 0.147 |
| Yes | 114 (16.7) | 226 (16.9) | 5 (12.2) | 10 (33.3) | 0 (0) | 355 (17) |  |
| No | 568 (83.3) | 1110 (83.1) | 36 (87.8) | 20 (66.7) | 2 (100) | 1736 (83) |  |
| Mental health |  |  |  |  |  |  | 0.077 |
| Yes | 106 (15.5) | 270 (20.2) | 7 (17.1) | 8 (26.7) | 0 (0) | 391 (18.7) |  |
| No | 578 (84.5) | 1065 (79.8) | 34 (82.9) | 22 (73.3) | 2 (100) | 1701 (81.3) |  |
| Family/interpersonal relations |  |  |  |  |  |  | <0.001 |
| Yes | 49 (7.2) | 170 (12.7) | 1 (2.6) | 6 (20) | 0 (0) | 226 (10.8) |  |
| No | 633 (92.8) | 1165 (87.3) | 38 (97.4) | 24 (80) | 2 (100) | 1862 (89.2) |  |
| Job/financial |  |  |  |  |  |  | 0.035 |
| Yes | 134 (19.6) | 333 (24.9) | 13 (31.7) | 9 (30) | 1 (50) | 490 (23.4) |  |
| No | 550 (80.4) | 1003 (75.1) | 28 (68.3) | 21 (70) | 1 (50) | 1603 (76.6) |  |
| Schooling |  |  |  |  |  |  | - |
| Yes | 22 (9.9) | - | - | - | - | 22 (9.9) |  |
| No | 201 (90.1) | - | - | - | - | 201 (90.1) |  |
| Daily life |  |  |  |  |  |  | 0.039 |
| Yes | 156 (22.8) | 338 (25.3) | 11 (26.8) | 14 (46.7) | 0 (0) | 519 (24.8) |  |
| No | 528 (77.2) | 996 (74.7) | 30 (73.2) | 16 (53.3) | 2 (100) | 1572 (75.2) |  |

Note: All the above-mentioned data were weighted; The data of no response were excluded for analysis.

**Table S4.** Reported COVID-19 stressors by occupation (N=2094)

|  |  | | | | | | | | | |  |
| --- | --- | --- | --- | --- | --- | --- | --- | --- | --- | --- | --- |
|  | White collar  (n=410) | Government employee  (n=86) | | Blue collar  (n=478) | | Shop owner/ business investor  (n=96) | | Professionals  (n=44) | Student  (n=224) | |  |
|  | n (%) | n (%) | | n (%) | | n (%) | | n (%) | n (%) | |  |
| Physical health |  |  | |  | |  | |  |  | |  |
| Yes | 81 (19.9) | 14 (16.1) | | 62 (13) | | 17 (17.5) | | 14 (31.8) | 24 (10.7) | |  |
| No | 327 (80.1) | 73 (83.9) | | 416 (87) | | 80 (82.5) | | 30 (68.2) | 200 (89.3) | |  |
| Mental health |  |  | |  | |  | |  |  | |  |
| Yes | 83 (20.2) | 10 (11.6) | | 92 (19.2) | | 27 (27.8) | | 9 (20.5) | 17 (7.6) | |  |
| No | 327 (79.8) | 76 (88.4) | | 386 (80.8) | | 70 (72.2) | | 35 (79.5) | 206 (92.4) | |  |
| Family/interpersonal |  |  | |  | |  | |  |  | |  |
| Yes | 44 (10.7) | 4 (4.7) | | 51 (10.7) | | 13 (13.4) | | 1 (2.3) | 11 (5) | |  |
| No | 366 (89.3) | 82 (95.3) | | 425 (89.3) | | 84 (86.6) | | 43 (97.7) | 211 (95) | |  |
| Job/financial |  |  | |  | |  | |  |  | |  |
| Yes | 100 (24.4) | 5 (5.8) | | 143 (29.9) | | 48 (49.5) | | 10 (22.7) | 21 (9.4) | |  |
| No | 310 (75.6) | 81 (94.2) | | 335 (70.1) | | 49 (50.5) | | 34 (77.3) | 203 (90.6) | |  |
| Schooling |  |  | |  | |  | |  |  | |  |
| Yes | - | - | | - | | - | | - | 22 (9.9) | |  |
| No | - | - | | - | | - | | - | 201 (90.1) | |  |
| Daily life |  |  | |  | |  | |  |  | |  |
| Yes | 116 (28.3) | 14 (16.3) | | 112 (23.4) | | 31 (32) | | 17 (38.6) | 42 (18.8) | |  |
| No | 294 (71.7) | 72 (83.7) | | 366 (76.6) | | 66 (68) | | 27 (61.4) | 181 (81.2) | |  |
| (continued) |  |  | |  | |  | |  |  | |  |
|  | Housewife  (n=307) | | Retired  (n=319) | | Jobless  (n=79) | | Freelancer  (n=51) | Total | |  | |
| Physical health |  | |  | |  | |  |  | | 0.004 | |
| Yes | 58 (18.9) | | 62 (19.5) | | 14 (17.5) | | 11 (21.6) | 357 (17) | |  | |
| No | 249 (81.1) | | 256 (80.5) | | 66 (82.5) | | 40 (78.4) | 1737 (83) | |  | |
| Mental health |  | |  | |  | |  |  | | <0.001 | |
| Yes | 60 (19.5) | | 75 (23.6) | | 11 (13.9) | | 7 (13.5) | 391 (18.7) | |  | |
| No | 247 (80.5) | | 243 (76.4) | | 68 (86.1) | | 45 (86.5) | 1703 (81.3) | |  | |
| Family/interpersonal |  | |  | |  | |  |  | | 0.006 | |
| Yes | 46 (15) | | 43 (13.5) | | 9 (11.4) | | 5 (9.8) | 227 (10.9) | |  | |
| No | 261 (85) | | 275 (86.5) | | 70 (88.6) | | 46 (90.2) | 1863 (89.1) | |  | |
| Job/financial |  | |  | |  | |  |  | | <0.001 | |
| Yes | 67 (21.8) | | 52 (16.4) | | 28 (35) | | 17 (33.3) | 491 (23.4) | |  | |
| No | 240 (78.2) | | 266 (83.6) | | 52 (65) | | 34 (66.7) | 1604 (76.6) | |  | |
| Schooling |  | |  | |  | |  |  | | - | |
| Yes | - | | - | | - | | - | 22 (9.9) | |  | |
| No | - | | - | | - | | - | 201 (90.1) | |  | |
| Daily life |  | |  | |  | |  |  | | 0.031 | |
| Yes | 78 (25.3) | | 75 (23.7) | | 21 (26.9) | | 13 (25) | 519 (24.8) | |  | |
| No | 230 (74.7) | | 242 (76.3) | | 57 (73.1) | | 39 (75) | 1574 (75.2) | |  | |
| Note: All Note: All the above-mentioned data were weighted; The data of no response were excluded for analysis. | | | | | | | | | | | |

**Table S5**. Pearson’s correlation matrix for major COVID-19 stressors, physical and psychological health measurements

|  | Physical health | Mental health | Family/  Interpersonal  stressor | Economic stressor | Daily life  stressor | BSRS-5  total scores | Suicide ideation | Loneliness | Self-efficacy |
| --- | --- | --- | --- | --- | --- | --- | --- | --- | --- |
| -Physical health | 1 | 0.493*** | 0.334*** | 0.252*** | 0.391*** | 0.227*** | 0.037 | 0.132*** | -0.135*** |
| -Mental health | 0.493*** | 1 | 0.313*** | 0.280*** | 0.451*** | 0.275*** | 0.079*** | 0.177*** | -0.154*** |
| Stressors: |  |  |  |  |  |  |  |  |  |
| -Family/  interpersonal | 0.334*** | 0.313*** | 1 | 0.320*** | 0.359*** | 0.237*** | 0.111*** | 0.198*** | -0.114*** |
| -Job/financial | 0.252*** | 0.280*** | 0.320*** | 1 | 0.385*** | 0.205*** | 0.036 | 0.132*** | -0.150*** |
| -Daily life | 0.391*** | 0.451*** | 0.359*** | 0.385*** | 1 | 0.239*** | 0.083*** | 0.173*** | -0.156*** |
| -BSRS5-5  total scores | 0.227*** | 0.275*** | 0.237*** | 0.205*** | 0.239*** | 1 | 0.309*** | 0.423*** | -0.253*** |
| -Suicide idea | 0.037 | 0.079*** | 0.111*** | 0.036 | 0.083*** | 0.309*** | 1 | 0.190** | -0.089*** |
| -Loneliness | 0.132*** | 0.177*** | 0.198*** | 0.132*** | 0.173*** | 0.423*** | 0.190*** | 1 | -0.188*** |
| -Self-efficacy | 0.135*** | 0.154*** | -0.114*** | -0.150*** | -0.156*** | -0.253*** | -0.089*** | -0.188*** | 1 |
| Note: All the above-mentioned data were weighted; missing data were excluded for analysis. **p<0.01, ***p<0.001.  Self-efficacy was categorized into three levels: low=0 (0-79), moderate=1 (80-85), high=2 (86-100);  BSRS-5: The Five-item Brief Symptom Rating Scale assessing psychiatric morbidity or psychological distress. | | | | | | | | | |
